# Supplementary material for: Immiscible hydrocarbon fluids in the deep carbon cycle
Source: Nat Commun. 2017 Jun 12;8:15798. doi: 10.1038/ncomms15798 (PMC5472781; doi:10.1038/ncomms15798)
Supplement: Supplementary Information [file ncomms15798-s1.pdf]

Type of file: PDF
Size of file: 0 KB
Title of file for HTML: Supplementary Information
Description: Supplementary Figures, Supplementary Tables and Supplementary References

Type of file: PDF
Size of file: 0 KB
Title of file for HTML: Peer Review File
Description:

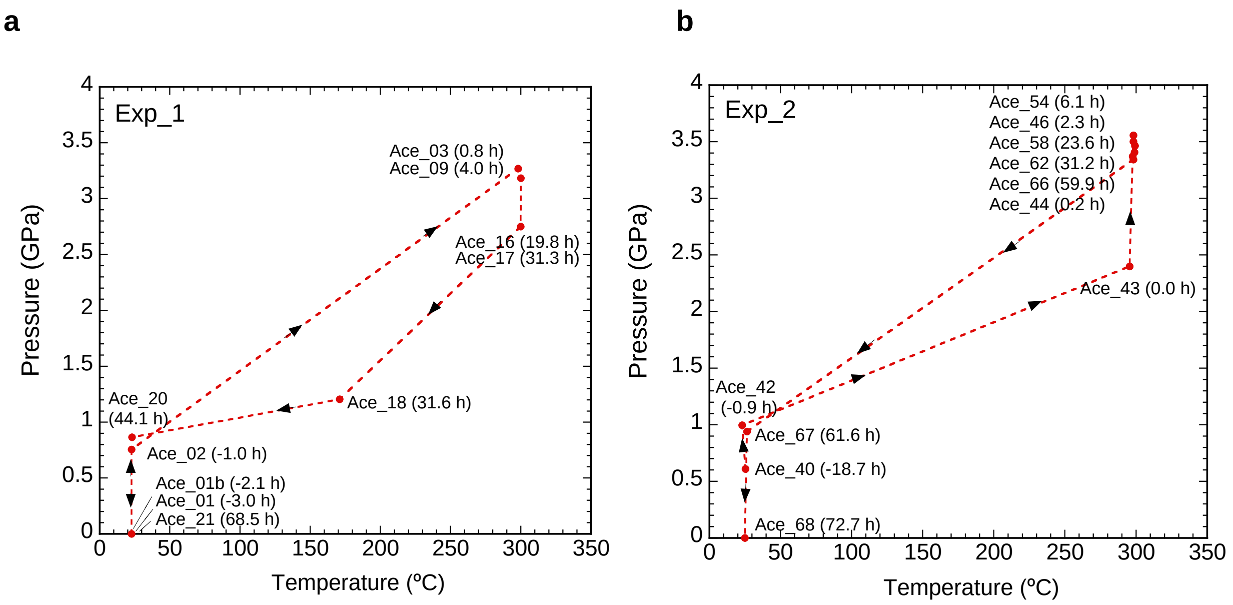

Supplementary Figure 1. Pressure-temperature paths of the diamond anvil experiments: (a) Exp_1; (b) Exp_2. The names of the measurements are listed besides the data points with numbers in parentheses indicating the time after heating of the measurement.

a

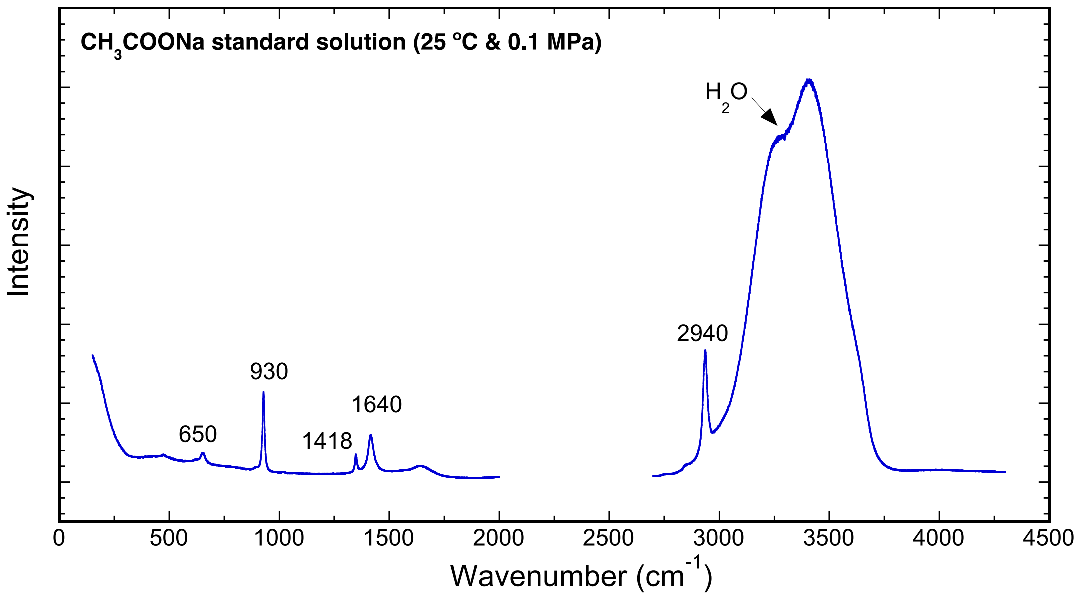

b

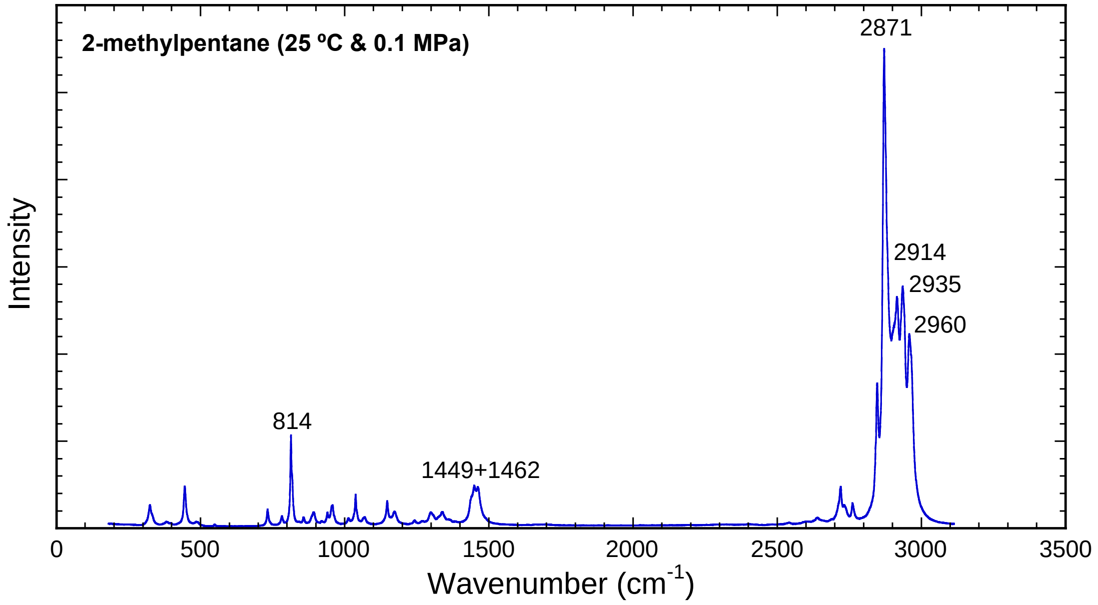

Supplementary Figure 2. Raman spectra of organic standards: (a) Sodium acetate solution used for experiments; (b) Standard spectra of pure liquid 2-methylpentane measured in the present study.

a

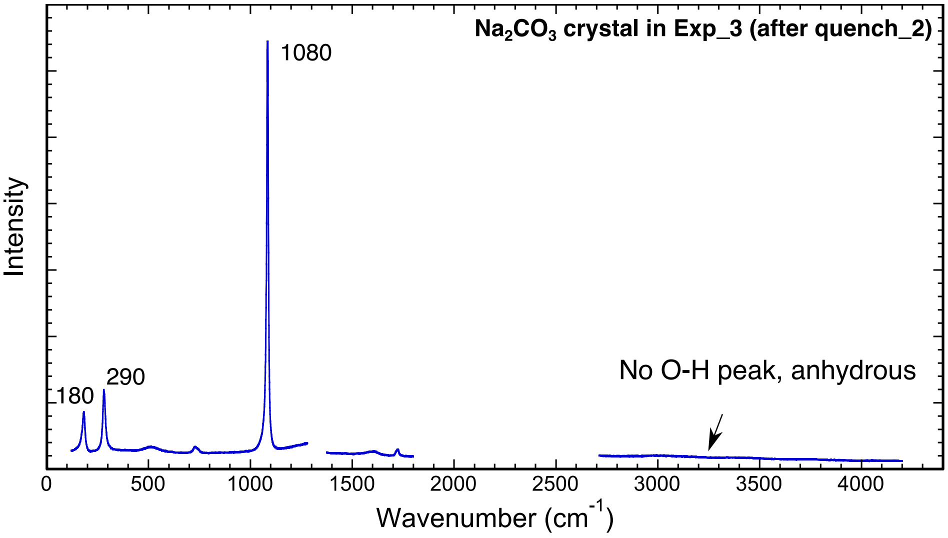

b

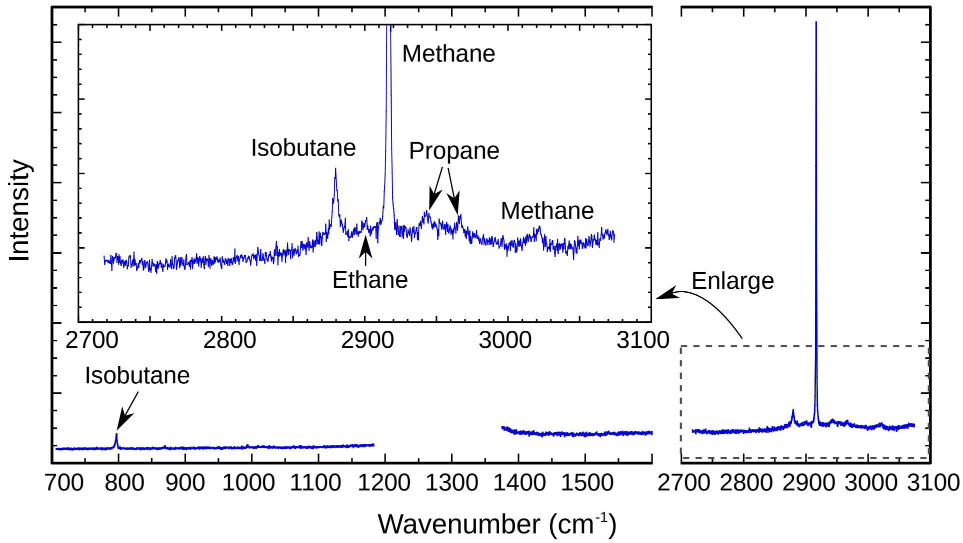

Supplementary Figure 3. Raman spectra of the species after quench_2: (a) An anhydrous Na_2CO_3 crystal according to previous experimental studies[3]; (b) A gas bubble.

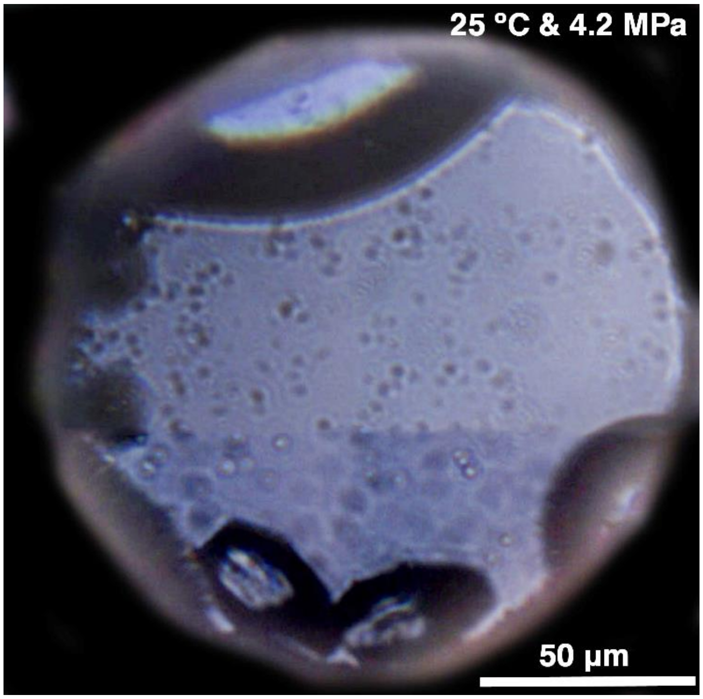

Supplementary Figure 4. Optical picture of the cell after quench_2. The gas bubbles are at the top and right bottom. The crystals are at the bottom, and the liquid hydrocarbon droplets are distributed all over the cell.

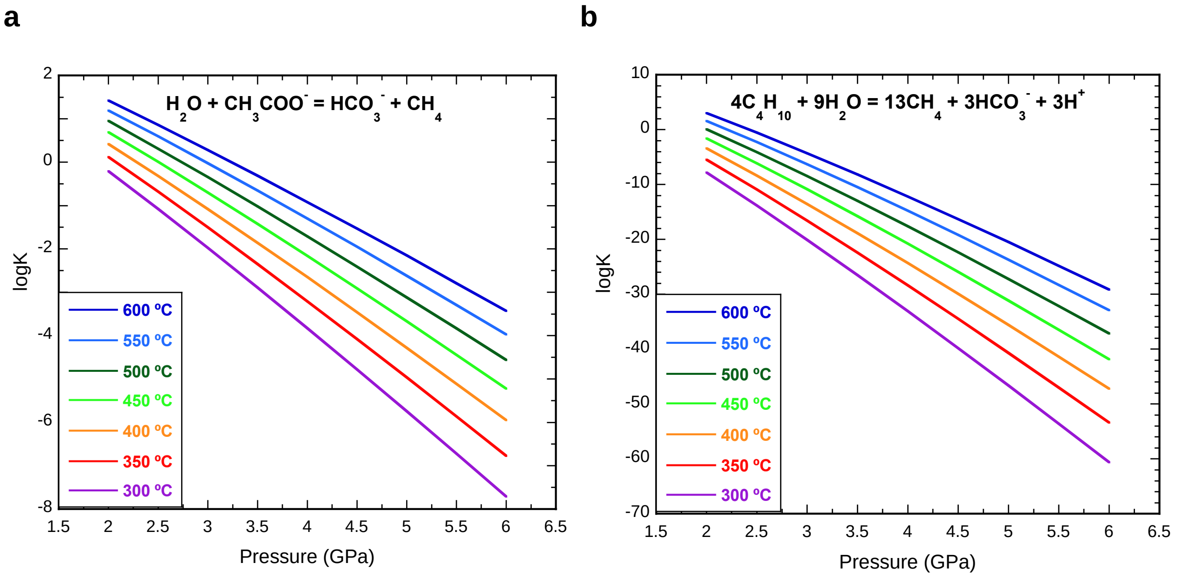

Supplementary Figure 5. Theoretical prediction of the log K values of two decomposition reactions using the DEW model: (a) Acetate decarboxylation to methane and bicarbonate; (b) Aqueous normal butane reaction into methane and bicarbonate. Due to a lack of thermodynamic data, we used normal butane to approximate isobutane. The latter should be even more stable than normal butane. Temperature and pressure ranges of both calculations are from 300 to 600 ºC and 2.0 GPa to 6.0 GPa.

Supplementary Table 1. Experimental conditions.

Starting materials	0.95 mol/L sodium acetate solution			
Temperature	300 ºC			
	Pressure (GPa)	Duration at elevated P-T (hours)	Laser during experiment	Material of gasket liner
Exp_1	3.1 (0.23)*	31	Yes	Pt
Exp_2	3.4 (0.07)*	60	Yes	Pt
Exp_3	3.1[#]	62	No	Pt
Exp_4	2.4[#]	0.8	Yes	Pt
Exp_5	3.5[#]	4	Yes	Pt
Exp_6	3.1 (0.08)*	28	Yes	Au

***The first number is the average of measured pressure during the experiment. The number in parentheses is the standard error.

[#]No parenthesis means only one pressure measurement was made.

Supplementary Table 2. Spectral wavenumbers and assignments for hydrocarbons.

	Methane[4,5]	Ethane[4,6]	Propane[4,5,7]	Isobutane[8]	2-methylpentane[9]	Droplet
C-H Stretching			2971			
		2960	2960	2958	2962	2965
		2942				2942*
		2925	2929	2933	2934	2936
	2916		2910	2907	2913	2913
		2885	2887	2889		2893
				2869	2873	2871
		2767	2771	2783		2777
		2734		2718	2822	2718
C-H Rocking	1534					
		1468		1468	1462	1464
			1451	1450	1444	1449
C-C Stretching		1190		1184	1173	1184
			1152	1169	1149	1170
			1054		1071	1068#
					1016	1020#
		998				996
		982		966	961	965
					936	933#
			922	917		
			869		890	870
					814	811
				799		800
			748		785	760
					732	730

*****A shoulder peak;

#The 933 cm^{-1}, 1020 cm^{-1} and 1068 cm^{-1} peaks are a mixture of peaks of organic species and the acetate, HCO_3^- and CO_3^{2-} peaks respectively, because spectra of aqueous organic species and the immiscible hydrocarbon species were recorded.

Supplementary Table 3. Calculated carbon content of immiscible hydrocarbons.

Exp_3 (Pt liner)				
**Time**	**Measured areas (pixel²)**		**Isobutane volume (%)***	**Carbon content (%)**
15 h	Droplet (most)	10193	1.68 (0.11)#	41.86 (2.76)#
	Droplet (least)	8930		
	Cell	567971		
21.6 h (a)	Droplet (most)	5136	1.59 (0.12)	39.61 (2.95)
	Droplet (least)	4424		
	Cell	300024		
21.6 h (b)	Droplet (most)	5233	1.57 (0.10)	39.16 (2.52)
	Droplet (least)	4601		
	Cell	312194		
62.2 h (quench_1)	Droplet (most)	12719	2.11 (0.13)	52.40 (3.29)
	Droplet (least)	11218		
	Cell	567971		
66.9 h (quench_2)	Droplet (most)	11061	1.81 (0.14)	43.84 (3.37)
	Droplet (least)	9483		
	Cell	567971		
87.5 h	Droplet (most)	12062	2.00 (0.16)	45.12 (3.57)
	Droplet (least)	10294		
	Cell	559256		
**Exp_5 (Pt liner)**				
**Time**	**Measured areas (pixel²)**		**Isobutane volume (%)***	**Carbon content (%)**
2.9 h	Droplet (most)	3946	0.67 (0.04)#	15.02 (0.90)#
	Droplet (least)	3498		
	Cell	559256		
**Exp_6 (Pt liner)**				
**Time**	**Measured areas (pixel²)**		**Isobutane volume (%)***	**Carbon content (%)**
0.3 h	Droplet (most)	4661	0.68 (0.06)#	16.93 (1.39)#
	Droplet (least)	3950		
	Cell	632172		
0.7 h	Droplet (most)	4732	0.70 (0.04)	17.52 (1.0)
	Droplet (least)	4175		
	Cell	632172		
1.9 h	Droplet (most)	4877	0.71 (0.06)	17.60 (1.53)
	Droplet (least)	4095		
	Cell	632172		
3.5 h	Droplet (most)	8886	1.33 (0.08)	32.87 (2.00)
	Droplet (least)	7868		
	Cell	632172		
4.5 h	Droplet (most)	10367	1.56 (0.08)	37.71 (2.04)
	Droplet (least)	9303		
	Cell	632172		
6.1 h	Droplet (most)	12163	1.81 (0.11)	44.90 (2.82)
	Droplet (least)	10723		
	Cell	632172		
8 h	Droplet (most)	12488	1.90 (0.08)	47.02 (1.98)
	Droplet (least)	11481		
	Cell	632172		
23.7 h	Droplet (most)	13163	1.97 (0.12)	48.75 (2.89)
	Droplet (least)	11689		
	Cell	632172		
25 h	Droplet (most)	13528	2.03 (0.11)	50.23 (2.85)
	Droplet (least)	12075		
	Cell	632172		
27.2 h	Droplet (most)	13468	2.01 (0.12)	49.88 (2.96)
	Droplet (least)	11960		
	Cell	632172		

*The isobutane volume percentage was calculated by dividing the area of droplets by the area of the cell. Then the area percentage was converted into a volume percentage.

[#]The number in the parentheses represents the standard error (see Methods).

Supplementary Table 4. The starting aqueous fluids and mineral assemblages, and the final compositions of each system at equilibrium at 300 ºC & 3.0 GPa.

**Starting Fluid (mol/kg)**	
CO_3^{2-}	0.1
CH_3COO^-	1
Cl^-	0.1
Na^+	0.01
K^+	0.01
Ca^{2+}	1.00E-06
Mg^{2+}	1.00E-06
Fe^{2+}	1.00E-12
Al^{3+}	1.00E-12
SiO_2(aq)	1.00E-06
pH	Charge balance

**Starting Pelitic (mol)**	
Quartz	0.6
Phlogopite	0.02
Annite	0.03
Muscovite	0.05
Albite	0.05
Anorthite	0.05

**Starting Mafic (mol)**	
Forsterite	0.05
Fayalite	0.05
Diopside	0.04
Hedenbergite	0.04
Albite	0.1
Anorthite	0.1

**Starting Ultramafic (mol)**	
Forsterite	0.18
Fayalite	0.02
Diopside	0.036
Hedenbergite	0.004
Enstatite	0.18
Ferrosilite	0.02
Clinochlore	0.02

**Final Pelitic (mmol)**	
Diaspore	207.1
Lawsonite	47.9
Muscovite	1.2
Coesite	861.5
Na_2CO_3	7.6
Phlogopite	0.5
Annite	0.5
Almandine	14.1
Pyrope	0.4
Grossular	0.1
Calcite	0.1
Magnesite	16.8
Siderite	15.2
Methane	***18***
Isobutane	***3.9***

**Final Mafic (mmol)**	
Lawsonite	150
Talc	20.4
Coesite	155.6
Na-carbonate	30.5
Ferrosilite	73.4
Enstatite-OR	15.2
Calcite	0.1
Magnesite	11.4
Siderite	16.3
Methane	***28.3***
Isobutane	***4.3***

**Final Ultramafic (mmol)**	
Antigorite	4.9
Talc	39
Clinochlore	20
Ferrosilite	8.5
Enstatite-OR	2.7
Calcite	2.6
Magnesite	38.3
Siderite	35.4
Methane	***41.6***
Isobutane	***3.1***

References.

1 Syracuse, E. M., van Keken, P. E. & Abers, G. A. The global range of subduction zone thermal models. *Physics of the Earth and Planetary Interiors* **183**, 73-90 (2010).

2 Li, Y. Immiscible C-H-O fluids formed at subduction zone conditions. *Geochemical Pespectives Letters* **3**, 12-21 (2017).

3 Buzgar, N. & Apopei, A. I. The Raman study of certain carbonates. *Analele Stiintifice de Universitatii AI Cuza din Iasi. Sect. 2, Geologie* **55**, 97 (2009).

4 Kolesnikov, A., Kutcherov, V. G. & Goncharov, A. F. Methane-derived hydrocarbons produced under upper-mantle conditions. *Nature Geoscience* **2**, 566-570 (2009).

5 Magnotti, G., KC, U., Varghese, P. & Barlow, R. Raman spectra of methane, ethylene, ethane, dimethyl ether, formaldehyde and propane for combustion applications. *Journal of Quantitative Spectroscopy and Radiative Transfer* **163**, 80-101 (2015).

6 Korppi-Tommola, J., Sundius, T., Shurvell, H. & Daunt, S. Multiple vibrational resonances in the Raman spectra of liquid ethanes. *Journal of Raman spectroscopy* **21**, 255-262 (1990).

7 Flurry, R. Vibrational assignments for propane from the nonrigid molecular symmetry group. *Journal of Molecular Spectroscopy* **56**, 88-92 (1975).

8 Evans, J. & Bernstein, H. The Vibrational Spectra of Isobutane and Isobutane-d 1. *Canadian Journal of Chemistry* **34**, 1037-1045 (1956).

9 Cleveland, F. F. & Porcelli, P. Raman Spectra of Hydrocarbons. V. n-Hexane, n-Heptane, 2-Methylpentane, 3-Methylpentane, 2, 4-Dimethylpentane, and 2, 3-Dimethylbutane. *The Journal of Chemical Physics* **18**, 1459-1461 (1950).
